# Adaptations of *Alteromonas* sp. 76-1 to Polysaccharide Degradation: A CAZyme Plasmid for Ulvan Degradation and Two Alginolytic Systems

**DOI:** 10.3389/fmicb.2019.00504

**Published:** 2019-03-18

**Authors:** Hanna Koch, Heike M. Freese, Richard L. Hahnke, Meinhard Simon, Matthias Wietz

**Affiliations:** ^1^Institute for Chemistry and Biology of the Marine Environment, University of Oldenburg, Oldenburg, Germany; ^2^Leibniz Institute DSMZ – German Collection of Microorganisms and Cell Cultures, Braunschweig, Germany

**Keywords:** alginate, ulvan, polysaccharide utilization loci, unique genes, niche specialization

## Abstract

Studying the physiology and genomics of cultured hydrolytic bacteria is a valuable approach to decipher the biogeochemical cycling of marine polysaccharides, major nutrients derived from phytoplankton and macroalgae. We herein describe the profound potential of *Alteromonas* sp. 76-1, isolated from alginate-enriched seawater at the Patagonian continental shelf, to degrade the algal polysaccharides alginate and ulvan. Phylogenetic analyses indicated that strain 76-1 might represent a novel species, distinguished from its closest relative (*Alteromonas naphthalenivorans*) by adaptations to their contrasting habitats (productive open ocean vs. coastal sediments). Ecological distinction of 76-1 was particularly manifested in the abundance of carbohydrate-active enzymes (CAZymes), consistent with its isolation from alginate-enriched seawater and elevated abundance of a related OTU in the original microcosm. Strain 76-1 encodes multiple alginate lyases from families PL6, PL7, PL17, and PL18 largely contained in two polysaccharide utilization loci (PUL), which may facilitate the utilization of different alginate structures in nature. Notably, ulvan degradation relates to a 126 Kb plasmid dedicated to polysaccharide utilization, encoding several PL24 and PL25 ulvan lyases and monomer-processing genes. This extensive and versatile CAZyme repertoire allowed substantial growth on polysaccharides, showing comparable doubling times with alginate (2 h) and ulvan (3 h) in relation to glucose (3 h). The finding of homologous ulvanolytic systems in distantly related *Alteromonas* spp. suggests CAZyme plasmids as effective vehicles for PUL transfer that mediate niche gain. Overall, the demonstrated CAZyme repertoire substantiates the role of *Alteromonas* in marine polysaccharide degradation and how PUL exchange influences the ecophysiology of this ubiquitous marine taxon.

## Introduction

Polysaccharides from phytoplankton and macroalgae represent a major fraction of organic matter in the oceans (Benner et al., 1992; Kraan, 2012; Rossi and De Philippis, 2016), especially in systems with high primary production such as continental shelves (Acha et al., 2004; Thomas et al., 2004). Algal polysaccharides are an important nutrient source for heterotrophic bacteria, as demonstrated by molecular studies of bacterial hydrolysis rates (Arnosti et al., 2011), population dynamics (Wietz et al., 2015), functional genomics (Martinez-Garcia et al., 2012; Matos et al., 2016) and cellular uptake processes (Reintjes et al., 2017) associated with polysaccharide degradation.

Cultured bacterial strains are a vital resource to study the genomic and ecophysiological basis of polysaccharide degradation, providing fundamental understanding of environmentally relevant processes. Culture-based studies were pivotal in revealing the functionality of carbohydrate-active enzymes (CAZymes) and their common localization in polysaccharide utilization loci (PUL), which allows the concerted degradation of polysaccharides (Hehemann et al., 2014; Grondin et al., 2017). To date, such studies have identified 322 families of polysaccharide lyases (PL), glycoside hydrolases (GH), carbohydrate-binding modules (CBM), carbohydrate esterases (CE), glycosyl transferases (GT) and auxiliary carbohydrate-active oxidoreductases (Lombard et al., 2014). CAZymes are commonly subject to horizontal gene transfer, the primary driver of strain-specific differences in hydrolytic capacities (Hehemann et al., 2016).

The continuously increasing number of sequenced bacterial genomes suggests that CAZymes are effective mechanisms of adaptation. For instance, the flavobacterium *Zobellia galactanivorans* is adapted to life on macroalgae through 50 PUL, providing numerous insights into PUL structure (Thomas et al., 2012), CAZyme biochemistry (Hehemann et al., 2012; Labourel et al., 2014), substrate-specific gene expression (Ficko-Blean et al., 2017; Thomas et al., 2017) and regulatory networks (Zhu et al., 2017). Comparable features in many *Bacteroidetes* (Mann et al., 2013; Kabisch et al., 2014) underline their predisposition toward polysaccharide degradation (Fernández-Gómez et al., 2013), but proficient hydrolytic capacities also occur among *Gammaproteobacteria* (Hehemann et al., 2017), *Verrucomicrobia* (Martinez-Garcia et al., 2012) and *Bacilli* (Zhu et al., 2016). Members of these taxa degrade a variety of polysaccharides produced by micro- and macroalgae, including laminarin, alginate, ulvan and pectin (Ekborg et al., 2005; Foran et al., 2017; Corzett et al., 2018).

To expand the understanding of bacterial CAZyme diversity and its role in ecological specialization, so-far understudied taxa can provide valuable insights. The gammaproteobacterial genus *Alteromonas* has been recently identified as important contributor to polysaccharide degradation in natural habitats, utilizing both dissolved (Wietz et al., 2015; Taylor and Cunliffe, 2017) and particulate substrates (Mitulla et al., 2016). Studies of model isolates have connected this functionality to diverse hydrolytic enzymes encoded in complex gene clusters (Chi et al., 2014; Neumann et al., 2015), whose expression is controlled by substrate availability (Koch et al., 2019). Although *Gammaproteobacteria* do not encode SusC/D proteins, the hallmark of PUL in *Bacteroidetes*, the finding of comparable functionality suggests these clusters can be designated PUL as well. Overall, model isolates provide conceptual understanding of ecophysiological adaptations that influence complex environmental processes, such as the succession of bacterial taxa during phytoplankton blooms (Teeling et al., 2012, 2016).

Here, we report the whole genome and physiology of a novel *Alteromonas* strain (designated 76-1) with pronounced potential for utilizing alginate and ulvan, structurally diverse polysaccharides that can constitute >50% of brown and green algae, respectively (Michel and Czjzek, 2013). Strain 76-1 has been isolated from an alginate-supplemented microcosm at the Patagonian continental shelf, a region with high primary productivity and hence regular availability of polysaccharides (Acha et al., 2004; Garcia et al., 2008). 16S rRNA gene amplicon sequencing showed that an OTU with 99% rRNA sequence identity was abundant in the original microcosm (Wietz et al., 2015), suggesting strain 76-1 as an environmentally relevant polysaccharide degrader. Genomic machineries for degradation of alginate and ulvan were compared to the closest relative (*Alteromonas naphthalenivorans* SN2^T^) and other *Alteromonas* strains, establishing an eco-evolutionary perspective into CAZyme-related niche specialization among *Alteromonas* and its connection to biogeochemical processes.

## Materials and Methods

### Isolation and Cultivation of Strain 76-1

*Alteromonas* sp. 76-1 was isolated in April 2012 from a microcosm with surface seawater collected at the Patagonian continental shelf (47° 56′41″S 61° 55′23″W) amended with 0.001% (w/v) sodium alginate (Wietz et al., 2015). Purity was confirmed by 16S rRNA gene PCR after several rounds of subculturing. Polysaccharide utilization was analyzed in seawater minimal medium (SWM) (Zech et al., 2009) supplemented with 0.1% sodium alginate (cat. no. A2158; Sigma-Aldrich, St. Louis, MO, United States) or 0.2% ulvan (cat. no. YU11689; Carbosynth, United Kingdom) as sole carbon sources in comparison to SWM + 0.1% glucose. All cultures were incubated in triplicates at 20°C and 100 rpm with regular photometric determination of growth (diluted if OD600 >0.4) followed by calculation of maximal growth rate (μmax) and doubling time (ln2/μmax). In addition, hydrolysis of 18 AZO-CL labeled polymers was tested according to Panschin et al. (2016). Briefly, AZO-CL substrates (Megazyme, Ireland) were distributed to individual microtiter wells in triplicate. To each well, 100 μL HaHa medium (Hahnke and Harder, 2013) were added, plus each 100 μL of a starved culture or 100 μL medium as control. Cultures were incubated at 25°C for up to 14 days and evaluated for hydrolytic activity on the basis of color change.

### Genome Sequencing and Taxonomy

Genomic DNA was extracted with the Genomic-tip 100/G kit (Qiagen, Hilden, Germany). After shearing using g-tubes (Covaris, Woburn, MA, United States) and monitoring the size range by pulse field gel electrophoresis, DNA fragments were end-repaired and ligated to hairpin adapters using P6 chemistry (Pacific Biosciences, Menlo Park, CA, United States). SMRT sequencing was carried out on a PacBio RSII instrument (Pacific Biosciences). PacBio reads were assembled *de novo* using the RS_HGAP_Assembly.3 protocol in the SMRT Portal v2.3. Indel errors were corrected using 5,759,952 paired-end reads of 112 bp from prior Illumina GAIIx sequencing on Nextera XT libraries (Illumina, San Diego, CA, United States) performed at Göttingen Genomics Laboratory (Germany). Illumina reads were mapped using the Burrows-Wheeler Aligner (Li and Durbin, 2009) followed by variant detection using VarScan v2.3.6 (Koboldt et al., 2012), consensus calling using GATK 3.1-1 (Mckenna et al., 2010), and trimming of the final assembly. Chromosome and plasmid were circularized (in total 4,817,656 bp) and uploaded to both ENA^1^ and IMG^2^ under accessions numbers PRJEB28726 and 2784132050, respectively. Phylogenetic analysis was carried out with 92 core genes identified using the UBCG pipeline (Na et al., 2018) including *Pseudoalteromonas atlantica* T6c as outgroup (RefSeq NC_008228.1). The concatenated nucleotide alignment was manually curated and the best substitution model (GTR+G) computed using jModelTest2 (Darriba et al., 2012). A maximum-likelihood phylogeny with 1000 bootstrap replicates was calculated using RaxML (Stamatakis, 2014) implemented on CIPRES (Miller et al., 2010).

### Comparative Genomics

Genomes of strain 76-1 and publicly available *Alteromonas* spp. (Supplementary Table S1) were compared using a variety of bioinformatic tools. Average amino acid identities and genome-to-genome distances were calculated using the Enveomics (Rodriguez-R and Konstantinidis, 2016) and GGDC (Meier-Kolthoff et al., 2013) web applications, respectively. Unique genes were identified using BPGA (Chaudhari et al., 2016) with a threshold of 50% amino acid identity, only considering proteins >50 amino acids (Supplementary Table S2). CAZymes were identified using dbCAN2 (Zhang et al., 2018), only considering hits with *e* < 10^−23^ and >80% query coverage. Annotations of selected genes were checked using UniprotKB-Swissprot, KAAS and MEROPS databases (Moriya et al., 2007; The UniProt Consortium, 2017; Rawlings et al., 2018). Genomic regions were visualized using genoPlotR (Guy et al., 2010) and Circos (Krzywinski et al., 2009) followed by manual inspection. Homologous PUL in related strains were identified using MultiGeneBlast (Medema et al., 2013), only considering proteins with >50% amino acid identity and >50% sequence coverage. Completeness of draft genomes used for comparative analyses was estimated using CheckM (Parks et al., 2015).

### Phylogeny and Annotation of Polysaccharide Lyases

Polysaccharide lyases of strain 76-1 were compared to enzymes deposited in the curated databases CAZY (Lombard et al., 2014) and PDB (Berman et al., 2000). In addition, related sequences deposited at NCBI were identified by BLASTp, only considering hits with >90% query coverage and >60% amino acid identity. PL sequences were aligned using ProbCons (Do et al., 2005) followed by manual inspection. Protein substitution models (WAG+G+F best for all alignments) were calculated using Modeltest-NG^3^, an improved successor of ProtTest3 (Darriba et al., 2011). Phylogenies were computed using RaxML with 1000 bootstrap replicates (Stamatakis, 2014). To determine the occurrence of PL transcripts in marine habitats, one PL from each alginolytic system (alt76_01684, alt76_03417) was searched against 272 marine metatranscriptomes publicly available at IMG (Supplementary Table S3). Only a co-detection of PL homologs with >70% amino acid identity was considered positive.

### Test for Antibacterial Activity and Siderophore Production

Two grams of XAD-16 resin (Dow Chemical Company, Midland, MI, United States) were washed several times with dH_2_O and methanol, concentrated on a metal filter, resuspended in 100 mL dH_2_O, and autoclaved. Strain 76-1 was precultured in sea salt medium (4% Sigma sea salts, 0.3% casamino acids, and 0.4% glucose) for 16 h at 20°C. Preculture was inoculated at 2% (v/v) into 100 mL sea salt medium mixed with 100 mL XAD-16 solution and cultivated at 20°C and 100 rpm for 24 h. Resin was collected by filtration and extracted with methanol:dH_2_O (80:20). Extracts were concentrated in a rotary evaporator using a two-step vacuum (337 mbar, 100 mbar) and heating to 40°C. Concentrated extract was tested for antimicrobial activity in a well-diffusion assay (Hjelm et al., 2004) with *Alteromonas macleodii* D7 as target strain and methanol:dH_2_O (80:20) as negative control. Siderophore production was tested with sterile-filtered supernatant from overnight cultures in both iron-deplete and iron-replete minimal medium using a modified CAS assay (Schwyn and Neilands, 1987; Alexander and Zuberer, 1991). Positive and negative controls were deferoxamine mesylate and sterile medium, respectively.

## Results and Discussion

### General Characteristics of *Alteromonas* sp. 76-1

**FIGURE 1:**
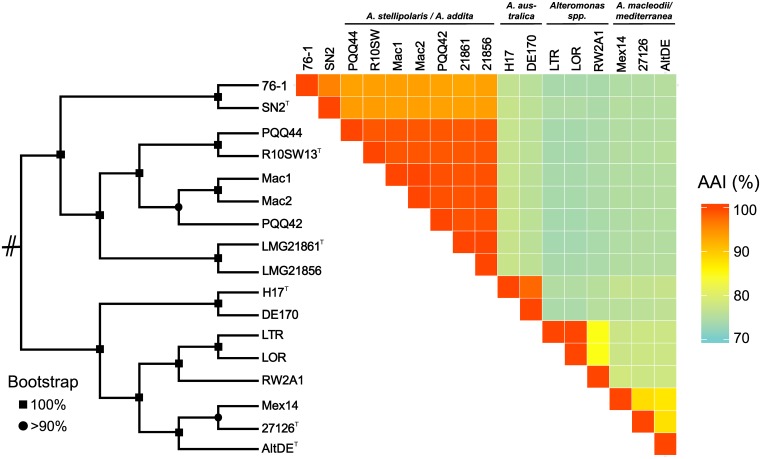
Taxonomic affiliation of *Alteromonas* sp. 76-1 based on maximum-likelihood core genome phylogeny and average amino acid identities (% AAI).

Strain 76-1 was isolated from alginate-supplemented seawater collected at the Patagonian continental shelf (Wietz et al., 2015) on solid media containing alginate as sole carbon source. Colonies appear round and cream-colored, assuming a tough surface after several days. Cells are motile as confirmed by light microscopy. Whole-genome sequencing revealed a chromosome of 4.7 Mb and a 126 Kb plasmid, encoding a total of 4100 proteins (Table 1). Core genome phylogeny demonstrated close relationship with *Alteromonas naphthalenivorans*, with 95.5% average amino acid identity (AAI) to *A. naphthalenivorans* SN2^*T*^ but only 63.5% DNA-DNA relatedness. Hence, strain 76-1 can only be assigned to genus level and might represent a novel *Alteromonas* species. Strains 76-1 and SN2 form a phylogenetic clade with *A. stellipolaris* and *A. addita* (89% AAI), separated from the more distant relatives *A. australica*, *A. macleodii*, and *A. mediterranea* (75–77% AAI) (Figure 1).

**Table 1:** Genome features of *Alteromonas* strains 76-1 and SN2.

Feature	76-1	SN2
Chromosome size (bp)	4,731,105	4,972,148
Plasmid size (bp)	125,985	not present
Average G+C content (%)	43.4	43.5
Protein-coding sequences (CDS)	4100	4335
rRNA genes	15	15
Strain-specific proteins^#^	621	793

*^#^based on 50% amino acid identity.*

**FIGURE 2:**
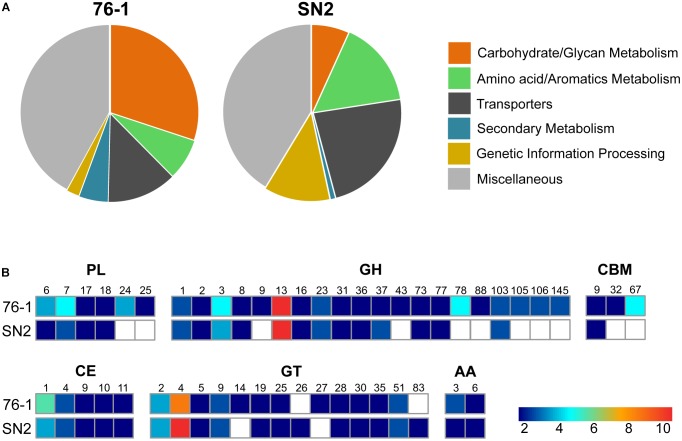
Genomic comparison of *Alteromonas* strains 76-1 and SN2. **(A)** Fraction of unique proteins assigned to specific KEGG classes. **(B)** Encoded CAZyme families, with numbers of enzymes from each family indicated by color (PL, polysaccharide lyase; GH, glycoside hydrolase; CBM, carbohydrate-binding module; CE, carbohydrate esterase; GT, glycosyl transferase; AA, auxiliary carbohydrate-active oxidoreductase).

The unclear taxonomic affiliation motivated an initial comparison of strain 76-1 with the closest relative, *A. naphthalenivorans* SN2^T^. In addition to a shared core of 3401 genes, strains 76-1 and SN2 encode 621 and 793 unique genes relating to their contrasting habitats (Table 1 and Supplementary Table S2). Notably, the origin of strain 76-1 from pelagic waters with high primary production and carbohydrate availability (Acha et al., 2004; Garcia et al., 2008) is reflected by a third of unique genes belonging to KEGG class “Carbohydrate Metabolism” (Figure 2A) and a diverse CAZyme repertoire (Figure 2B). Strain 76-1 encodes a total of 96 CAZymes, including thirteen polysaccharide lyases from six families and 41 glycoside hydrolases from 20 families (Table 2 and Figure 2B). This CAZyme content approximates that of algae-associated *Flavobacteriia* (Mann et al., 2013) whereas most other *Alteromonas* encode lower numbers (Supplementary Table S1).

Based on these observations, this study focuses on the genomics and ecophysiology of polysaccharide degradation, as detailed below, but 76-1 features further unique adaptations. Specifically, unique gene clusters for siderophore synthesis and methylamine metabolism (Supplementary Figure S1) could sustain iron and nitrogen supply under limiting conditions in pelagic habitats (Boiteau et al., 2016; Taubert et al., 2017). The siderophore cluster is co-localized with polyketide-nonribosomal peptide (PKS-NRPS)-encoding genes that may enhance competitive abilities (Patin et al., 2017). We experimentally confirmed functionality of these clusters by showing iron scavenging and antibacterial activity using CAS and well-diffusion assays (Supplementary Figure S1). Homology of the PKS-NRPS to tyrocidine/gramicidin-related gene clusters in *Bacillus* (Mootz and Marahiel, 1997) and differing G+C content compared to the chromosome (38 vs. 44%) suggest horizontal acquisition from low-GC Gram-positive bacteria. In contrast, proteins for naphthalene and urea degradation (Math et al., 2012; Jin et al., 2016) as well as conjugal transfer (Supplementary Table S2) are unique to *A. naphthalenivorans* SN2^T^ and probably facilitate adaptation to higher anthropogenic input (Cozzi et al., 2014) and genetic exchange (Fuchsman et al., 2017) in coastal sediments. The lower numbers and diversity of CAZymes (Figure 2) suggests a minor role of polysaccharide degradation in the habitat of SN2 (Math et al., 2012).

### Proficiency of *Alteromonas* sp. 76-1 to Degrade Different Algal Polysaccharides

A comprehensive CAZyme inventory of *Alteromonas* sp. 76-1 revealed genetic machineries for degradation of alginate, ulvan and xylan, common polysaccharides from marine algae (Michel and Czjzek, 2013) and potentially contained in algal-derived microgels (Aluwihare and Repeta, 1999). Furthermore, we identified functional genes for the degradation of different glucans with simpler molecular structure.

**FIGURE 3:**
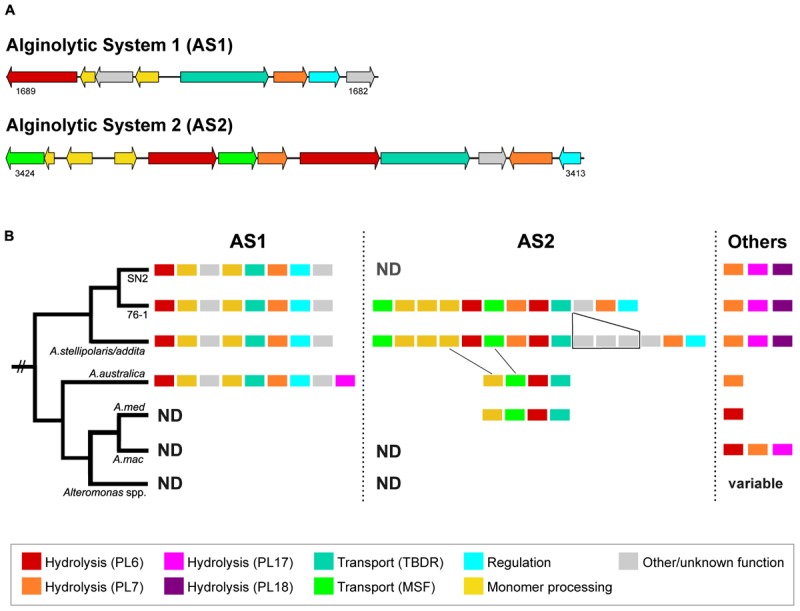
**(A)** Alginolytic systems AS1 and AS2 in *Alteromonas* sp. 76-1 (numbers designate IMG locus tags). **(B)** Schematic representation of AS1/2 and other alginate lyases in 76-1 and related *Alteromonas* strains. Alginate lyases in *A. macleodii* are organized in PUL with different structure and only schematically indicated here. Also, lyase numbers in *A. mediterranea* and more distantly related *Alteromonas* spp. vary between strains (see Neumann et al., 2015; Koch et al., 2019). PL, polysaccharide lyase; TBDR, TonB-dependent receptor; MSF, major facilitator superfamily transporter; ND, not detected. For detailed comparison, see Supplementary Figure S3.

Alginate degradation of strain 76-1 relates to two separate alginolytic systems (designated AS1 and AS2) encoding PL6 and PL7 alginate lyases, TonB-dependent receptors, MFS transporters and enzymes for monomer processing (Figure 3A). Additional alginate lyases from families PL7, PL17, and PL18 are located outside of AS1 and AS2 (Figure 3B and Supplementary Table S2). The role of these enzymes in alginate degradation is supported by distinct homologies to biochemically characterized alginate lyases (Table 3 and Supplementary Figure S2) and co-localization of genes for downstream processing. Alginate lyases encoded in AS1 are probably exolytic, including alt76_01689 with homology to a PL6 from *Paraglaciecola chathamensis* (Xu et al., 2017) and alt76_01684 with homology to a PL7 from *Zobellia galactanivorans* that is specifically upregulated in the presence of alginate (Thomas et al., 2017). AS2 encodes another exolytic candidate PL6 and two homologs of endolytic PL7 from *Vibrio splendidus* releasing trisaccharides (Lyu et al., 2018). In line with other studies, the separate PL17 (alt76_2604) probably degrades released oligomers, exemplified by distinct similarity to PL17_4NEI from *Saccharophagus degradans* (Park et al., 2014). The separate PL18 (alt76_00345) shares >50% amino acid similarity to PL18_4Q8L from *Pseudoalteromonas* sp. 0524 with complete conservation of the seven central residues, indicating endolytic release of di- and trisaccharides (Dong et al., 2014). The PL18 furthermore harbors two predicted CBMs from families 16 and 32, which may enable binding different alginate motifs (Sim et al., 2017) or contribute to protein maturation (Dong et al., 2014).

**Table 2:** CAZyme families encoded on chromosome and plasmid of *Alteromonas* sp. 76-1 as predicted by dbCAN2.

Class^#^	Families on chromosome	Families on plasmid
PL	6 7 17 18	24 25
GH	1 2 3 8 9 13 16 23 31 36 37 73 77 103	43 78 88 105 106 145
CBM	9 32	67
CE	1 4 9 10 11	
GT	2 4 5 9 14 19 25 27 28 30 35 51	
AA	3 6	

*^#^PL, polysaccharide lyase; GH, glycoside hydrolase; CBM, carbohydrate-binding module; CE, carbohydrate esterase; GT, glycosyl transferase; AA, auxiliary carbohydrate-active oxidoreductase.*

**FIGURE 4:**
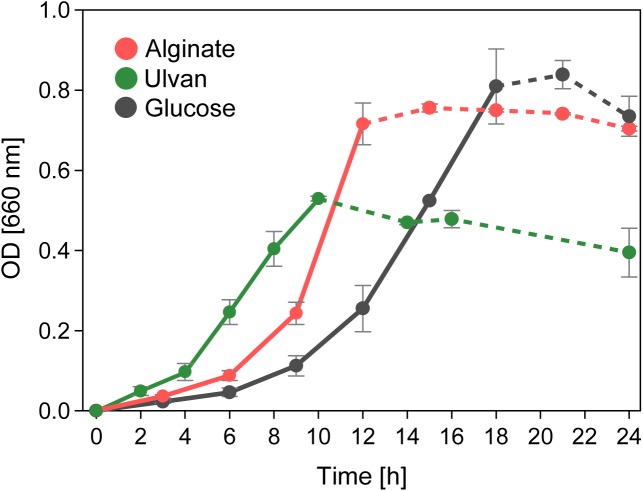
Alginolytic and ulvanolytic systems enable distinct growth of *Alteromonas* sp. 76-1 with alginate and ulvan as sole carbon source. Cell aggregates forming with the onset of stationary phase impeded reliable OD measurements (indicated by broken lines).

Accordingly, strain 76-1 grew proficiently with alginate as sole carbon source (Figure 4). Faster growth than with glucose was notable, as degradation of the complex alginate polymer (comprising heterogeneous blocks of mannuronate and guluronate) involves more enzymatic steps and induces a prolonged lag phase in other *Alteromonas* strains (Neumann et al., 2015). Utilization may be boosted by FabG-like enzymes encoded in both AS (Supplementary Figure S3) with homology to alginate-specific reductases in *Flavobacteriia* (Inoue et al., 2015). These enzymes possibly accelerate the central downstream conversion of 4-deoxy-L-erythro-5-hexoseulose uronate to 2-keto-3-deoxy-D-gluconate and hence prevent bacteriostasis from accumulation of metabolic intermediates (Fuhrman et al., 1998).

**Table 3:** Similarities of polysaccharide lyases of *Alteromonas* sp. 76-1 to biochemically characterized enzymes in the PDB database.

Gene (alt76_)	CAZyme family	PDB	Organism	% ID/coverage	Function	Reference
00345	PL18, CBM16, CBM32	4Q8L	*Pseudoalteromonas* sp. SM0524	54/41	endolytic alginate lyase (M-M, G-G)	Dong et al., 2014
01684	PL7	4BE3	*Zobellia galactanivorans*	36/88	exolytic alginate lyase (M-M, M-G, G-G)	Thomas et al., 2013
01689	PL6	5GKD	*Paraglaciecola chathamensis*	49/90	exolytic alginate lyase (G-G)	Xu et al., 2017
02604	PL17	4NEI	*Saccharophagus degradans*	39/99	cleaves oligosaccharide	Park et al., 2014
03213	PL7	4BE3	*Zobellia galactanivorans*	34/62	exolytic alginate lyase (M-M, M-G, G-G)	Thomas et al., 2013
03414	PL7, CBM32	5ZU5	*Vibrio splendidus*	51/57	alginate lyase (M-M and G-G)	Lyu et al., 2018
03417	PL6	nd	nd	nd	nd	nd
03418	PL7	5ZU5	*Vibrio splendidus*	53/82	alginate lyase (M-M and G-G)	Lyu et al., 2018
03420	PL6	5GKD	*Paraglaciecola chathamensis*	59/93	exolytic alginate lyase (G-G)	Xu et al., 2017
04143	PL24	6BYP	*Alteromonas* sp. LOR	46/92	ulvan lyase (Rha3S-GlcA)	Kopel et al., 2016; Ulaganathan et al., 2018
04144	PL24	6BYP	*Alteromonas* sp. LOR	88/95	ulvan lyase (Rha3S-GlcA)	Kopel et al., 2016; Ulaganathan et al., 2018
04173	PL25	5UAM	*Pseudoalteromonas* sp. PLSV	77/92	ulvan lyase (Rha3S-GlcA, Rha3S-IdoA)	Ulaganathan et al., 2017
04188	PL24	6BYP	*Alteromonas* sp. LOR	67/49	ulvan lyase (Rha3S-GlcA)	Kopel et al., 2016; Ulaganathan et al., 2018

*PL, polysaccharide lyase; CBM, carbohydrate-binding module; M, mannuronate; G, guluronate; Rha3S, 3-sulfated rhamnose; GlcA, glucuronic acid; IdoA, iduronic acid; nd, no homolog detected with >30% identity/query coverage.*

The alginolytic machinery of 76-1 is found in several *Alteromonas* strains, indicating a broader distribution of alginate degradation among this taxon than previously assumed. AS1 and the separate PL17/18 are conserved in the phylogenetic clade comprising strain 76-1, *A. naphthalenivorans*, *A. stellipolaris*, and *A. addita* (Figure 1 and Supplementary Figure S3). AS1 also occurs in the next related species (*A. australica*) but including the PL17 that is encoded separately in 76-1 (Figure 3B). AS2 shows greater structural variability between strains; with three genes including a putative TBDR plug domain (The UniProt Consortium, 2017) missing in 76-1 and a further reduced version in *A. australica* and some *A. mediterranea* (Figure 3B). Different PUL architectures and PL rearrangements are consistent with *Alteromonas* genome plasticity (López-Pérez et al., 2017) and may confer ecological differentiation, as lyase copy number correlates with enzymatic activity due to gene dosage effect (Hehemann et al., 2016). In the broader eco-evolutionary context, the alginolytic machinery of 76-1 might play an important role, allowing degradation of different alginate structures (Peteiro, 2018) and natural polysaccharide pulses that frequently occur at the productive Patagonian shelf (Acha et al., 2004; Romero et al., 2006). Accordingly, an OTU with 99% 16S rRNA gene similarity was abundant in the alginate-supplemented microcosm from which 76-1 was isolated (Wietz et al., 2015). The ecological relevance was underlined by co-detecting transcripts of alginate lyases from both AS near the original isolation site (Supplementary Table S3), suggesting active roles in natural habitats.

### A CAZyme Plasmid for Ulvan Degradation

**FIGURE 5:**
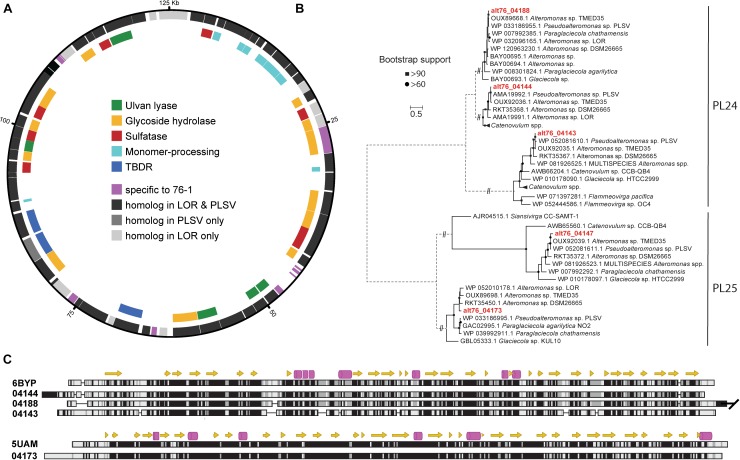
**(A)** CAZyme plasmid of *Alteromonas* sp. 76-1 encoding an ulvanolytic system. Outer circle: all genes; encompassing genes with homologs in *Pseudoalteromonas* sp. PLSV and/or *Alteromonas* sp. LOR (>50% amino acid identity/query coverage) or specific to strain 76-1. Inner circle: genes encoding CAZymes, sulfatases, TonB-dependent receptors (TBDR) and monomer-processing genes. **(B)** Maximum-likelihood phylogeny of PL24/PL25 protein sequences of 76-1 and homologs in CAZY and NCBI databases. **(C)** Structural alignment of PL24 and PL25 ulvan lyases with biochemically characterized homologs (Table 3). Alpha helices are shown as arrows and beta strands as barrels. Low-to-high similarities based on a Blosum62 matrix are indicated with a white-to-black gradient. Only the first 517 amino acids were aligned for alt76_04188, since the C-terminal extension is not conserved in the other analyzed PL24.

The plasmid of *Alteromonas* sp. 76-1 is markedly devoted to polysaccharide degradation (Figure 5A and Table 2). Twenty percent of plasmid genes encode CAZymes (plus additional sulfatase and monomer-processing genes) in comparison to 2% CAZymes on the chromosome. This CAZyme condensation essentially denotes the plasmid as an extrachromosomal PUL, matching the size of the largest PUL hitherto described in marine bacteria (Schultz-Johansen et al., 2018). Comparison to characterized CAZymes suggest that the plasmid is linked to degradation of ulvan, a common polysaccharide of green algae (Michel and Czjzek, 2013) mainly consisting of 3-sulfated rhamnose, iduronic acid, glucuronic acid and xylose. Accordingly, strain 76-1 showed distinct growth on ulvan as sole carbon source, with doubling times comparable to glucose (Figure 4). This activity relates to three PL24 and two PL25 ulvan lyases, multiple GHs including predicted rhamnosidases, as well as transporters and monomer-processing genes in the plasmid-encoded ulvanolytic system (Figure 5A). Highly homologous proteins in similar organization also occur in *Alteromonas* sp. LOR and *Pseudoalteromonas* sp. PLSV (Kopel et al., 2016; Foran et al., 2017), and several of these homologs have been characterized biochemically (Table 3). All encoded PL24 of strain 76-1 are homologous to the PL24_6BYP ulvan lyase from *Alteromonas* sp. LOR, although phylogenetic clustering of alt76_04143 with flavobacterial PLs suggests another evolutionary origin (Figure 5B). The proposed functionality of the ulvanolytic system is supported by structural alignments (Figure 5C), showing conservation in residues essential for endolytic ulvan cleavage (Ulaganathan et al., 2017, 2018). Activity assays in strains LOR and PLSV have illustrated potential routes of downstream degradation, showing release of two uronic tetrasaccharides by PL24_6BYP (Ulaganathan et al., 2018) which were in turn degraded by PL25_5UAM from strain PLSV (Qin et al., 2018). Considering sizeable genomic similarities and physiological evidence, we assume similar action by the homologous enzymes in 76-1.

Although regulatory mechanisms remain to be determined, the rapid initiation of growth indicates that the plasmid localization accelerates ulvan degradation, as plasmids are less affected by DNA supercoiling (Dorman and Dorman, 2016) and early-replicating regions (due to the proximity of lyases and origin of replication) are characterized by higher expression (Oliveira et al., 2017). Moreover, the common induction of PUL by substrate availability (Thomas et al., 2017; Koch et al., 2019) suggests that enzymes are only produced in presence of ulvan, reducing fitness costs and the risk of plasmid loss (MacLean and San Millan, 2015). Intriguingly, the encoding contigs in *Alteromonas* sp. LOR and *Pseudoalteromonas* sp. PLSV (estimated completeness of draft genomes >99%) harbor partitioning proteins with 83% identity to those on the 76-1 plasmid (alt76_04111–12), suggesting equivalent localization on plasmids acquired in separate horizontal transfer events. Strains LOR and PLSV originate from sea slugs feeding on ulvan-rich macroalgae, indicating ecological relevance of ulvan degradation in host-microbe interactions (Gobet et al., 2018a; Konasani et al., 2018). Isolation of LOR/PLSV from the Brittany coast and hence the opposite side of the Atlantic suggest that ulvanolytic plasmids are distributed over wide geographic scales and maintained in strains from certain niches. Although PUL on plasmids are known in marine bacteria (Zhong et al., 2001) this is only the second report of a plasmid almost fully dedicated to polysaccharide metabolism (Gobet et al., 2018b). Mobile CAZyme elements are likely an important eco-evolutionary factor, representing efficient vehicles of PUL that provide recipient strains with access to specific “polysaccharide niches” on short time scales (Hülter et al., 2017).

### Degradation of Other Polysaccharides

The predisposition of *Alteromonas* sp. 76-1 toward polysaccharides was underlined by testing hydrolysis of 18 AZO-CL labeled substrates (Panschin et al., 2016). Strain 76-1 hydrolyzed diverse glucans with α-(1–4), β-(1–3), and β-(1–4) bonds, including amylose, pullulan, pachyman and barley β-glucan (Supplementary Table S4). Amylase and pullulanase activity likely relates to a gene cluster with four GH13 enzymes plus an additional GH13 in a distant genomic location. Pachyman and barley β-glucan are probably hydrolyzed by the same endo-β-(1–3,4)-glucanase from family GH16. Commonly described xylanase families (GH10, GH30, GH67, and GH115) were not detected in the genome, but xylanolytic activity likely corresponds to a unique chromosomal cluster harboring a GH3 β-(1–4)-xylosidase, a GH9 and several xylose-processing genes (Supplementary Table S4). Proteolytic activity exemplified by casein hydrolysis demonstrated that 76-1 also utilizes other polymer types, attributed to 97 encoded peptidases and proteases predicted by MEROPS (Supplementary Table S2). However, this number is only about half compared to related *Alteromonas* such as *A. macleodii*, which in turn encode less CAZymes (Supplementary Table S1). This observation indicates differing preferences between *Alteromonas* lineages toward polysaccharides and proteins.

## Conclusion

The presence of diverse polysaccharide lyases, two alginolytic systems and a plasmid dedicated to ulvan degradation demonstrate a marked adaptation of *Alteromonas* sp. 76-1 toward polysaccharides from algae, potentially allowing considerable hydrolytic activity in marine systems. The plasmid-mediated adaptation to ulvan is unprecedented to date, illustrating that mobile PUL can provide access to certain “polysaccharide niches” and represent an important eco-evolutionary factor. It remains to be determined whether the predisposition of 76-1 toward algal polysaccharides corresponds to occurrence on macroalgae, although *Alteromonas* spp. are seldom reported as algal epibionts to date and may rather target polysaccharides derived from exudation or decay (Koch et al., 2019). Considering the substantial primary production of algae on global scales and the relevance of polysaccharides in marine food webs, these insights contribute to the understanding of niche specialization among marine *Alteromonas* and how their CAZyme repertoire influences the biogeochemical cycling of abundant algal polysaccharides.

## Author Contributions

MW, HK, and MS designed the research and wrote the manuscript. HK and MW conducted the genomic analyses and growth experiments. HF performed the complete genome assembly with error correction and annotation. RH conducted the AZO-CL assay. All authors contributed to the final version of the manuscript.

## Conflict of Interest Statement

The authors declare that the research was conducted in the absence of any commercial or financial relationships that could be construed as a potential conflict of interest.

## Footnotes

1 https://www.ebi.ac.uk/ena

2 https://img.jgi.doe.gov

3 https://github.com/ddarriba/modeltest

## Supplementary Material

FIGURE S1 Unique gene clusters in *Alteromonas* sp. 76-1. **(A)** hybrid biosynthetic gene cluster encoding a functional siderophore (insert depicting CAS assay including positive and negative control) and active antibacterial compound (insert depicting inhibition of *A. macleodii* D7 in well-diffusion agar assay; marked in yellow). **(B)** gene cluster for methylamine metabolism (mau, methylamine dehydrogenase; cyt, cytochrome). Numbers below clusters designate IMG locus tags.

FIGURE S2 Amino acid-based maximum-likelihood phylogeny of PL6 alginate lyases of *Alteromonas* sp. 76-1 (red label), related *Alteromonas* spp. and deposited sequences at the CAZY database. Only relevant subtrees are shown; labeled with locus tags/NCBI gene identifiers, strain and annotation.

FIGURE S3 Detailed overview of AS1 and AS2 in *Alteromonas* sp. 76-1 and related strains. PL, polysaccharide lyase; TBDR, TonB-dependent receptor; MSF, major facilitator superfamily transporter; KdgF, protein for uronate linearization; DehR, 4-deoxy-L-erythro-5-hexoseulose uronate (DEH) reductase; FabG, oxidoreductase with similarity to DEH reductase; Nramp, metal-ion transporter; HP, protein of unknown function. Numbers below clusters designate IMG locus tags.

TABLE S1 List of *Alteromonas* genomes analyzed, including gene and CAZyme count.

TABLE S2 Sheet Alt761_CDS: Complete list of protein-coding sequences of *Alteromonas* sp. 76-1 including CAZyme prediction using dbCAN2, KEGG annotation using KAAS, and protease/peptidase prediction using MEROPS. Sheet Uniques_Alt761_SN2: Unique genes and associated KEGG classifications in *Alteromonas* strains 76-1 and SN2, including CAZyme prediction using dbCAN2 and KEGG annotation using KAAS.

TABLE S3 Detection of transcripts homologous to alginate lyases PL7_AS1 (alt76_01684) and PL6_AS2 (alt76_03417) from *Alteromonas* sp. 76-1 in marine metatranscriptomes. Red label: co-detection of both transcripts in the same metatranscriptome. The picture insert illustrates origin of 76-1 and co-detection of alginate lyases in metatranscriptomes from nearby locations.

TABLE S4 Hydrolysis of AZO-CL polymers by *Alteromonas* sp. 76-1 and proposedly involved genes.
