# Supplementary figures and images for: Adaptations of Alteromonas sp. 76-1 to Polysaccharide Degradation: A CAZyme Plasmid for Ulvan Degradation and Two Alginolytic Systems
Source: Front Microbiol. 2019 Mar 18;10:504. doi: 10.3389/fmicb.2019.00504 (PMC6431674; doi:10.3389/fmicb.2019.00504)

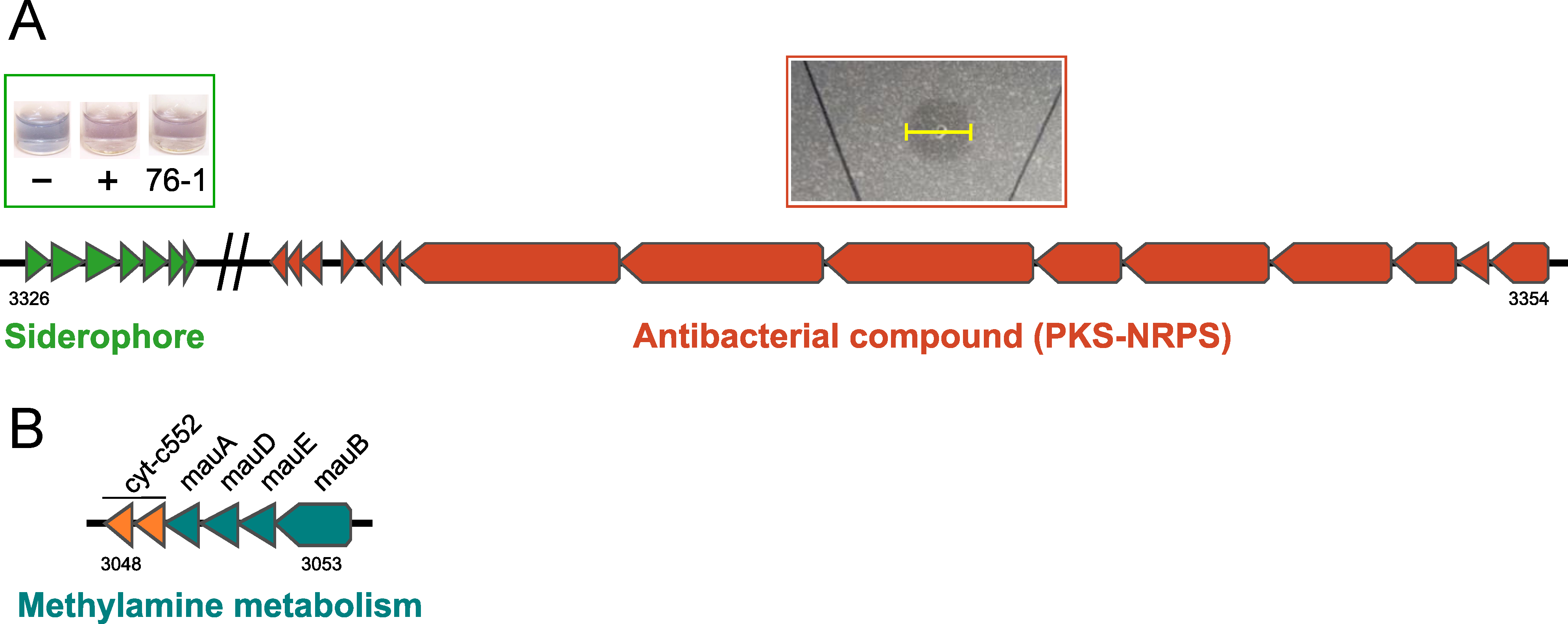

Supplement: FIGURE S1 — Unique gene clusters in Alteromonas sp. 76-1. (A) hybrid biosynthetic gene cluster encoding a functional siderophore (insert depicting CAS assay including positive and negative control) and active antibacterial compound (insert depicting inhibition of A. macleodii D7 in well-diffusion agar assay; marked in yellow). (B) gene cluster for methylamine metabolism (mau, methylamine dehydrogenase; cyt, cytochrome). Numbers below clusters designate IMG locus tags. [file Image_1.TIF]

# AS1

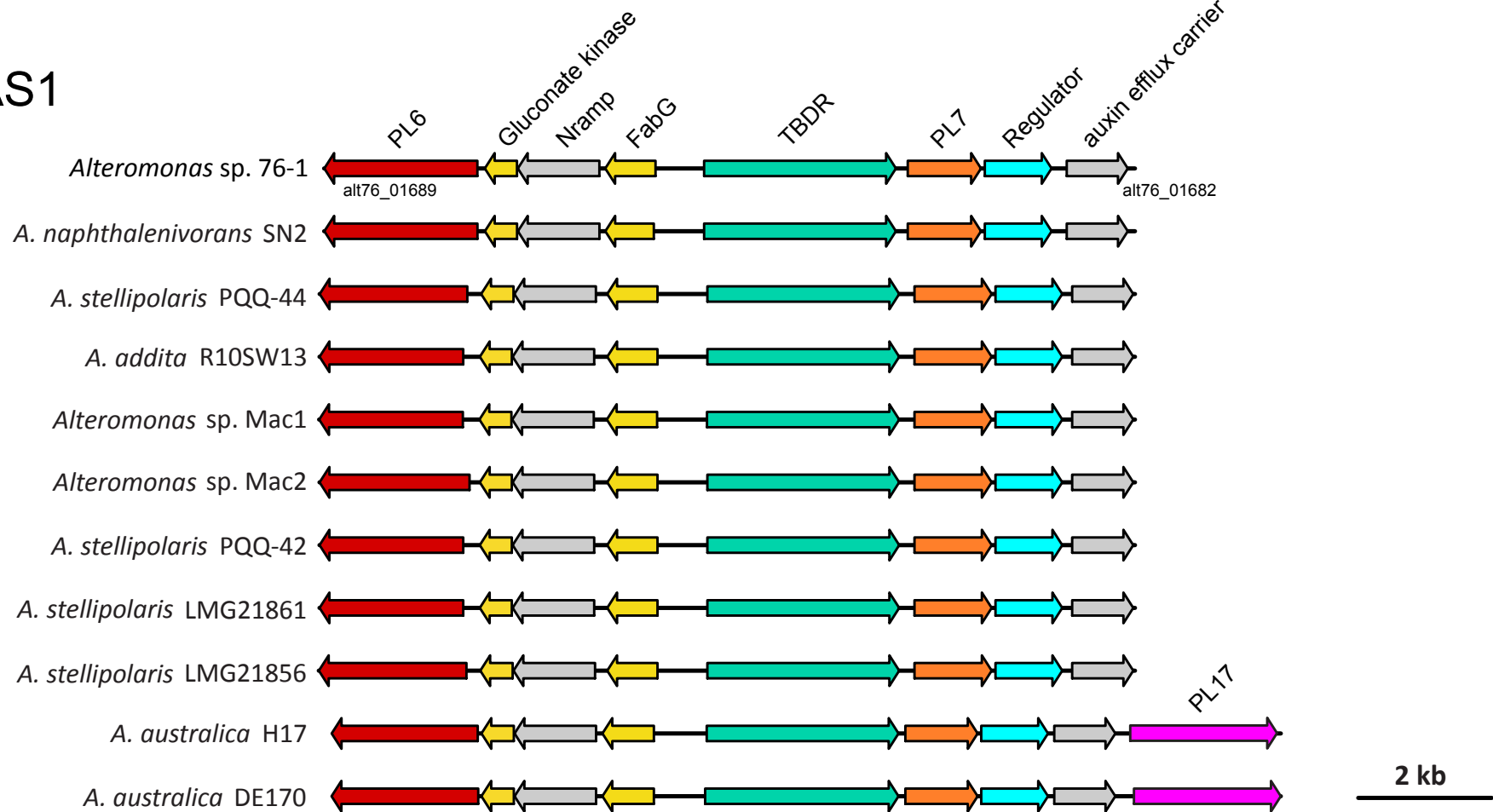

# AS2

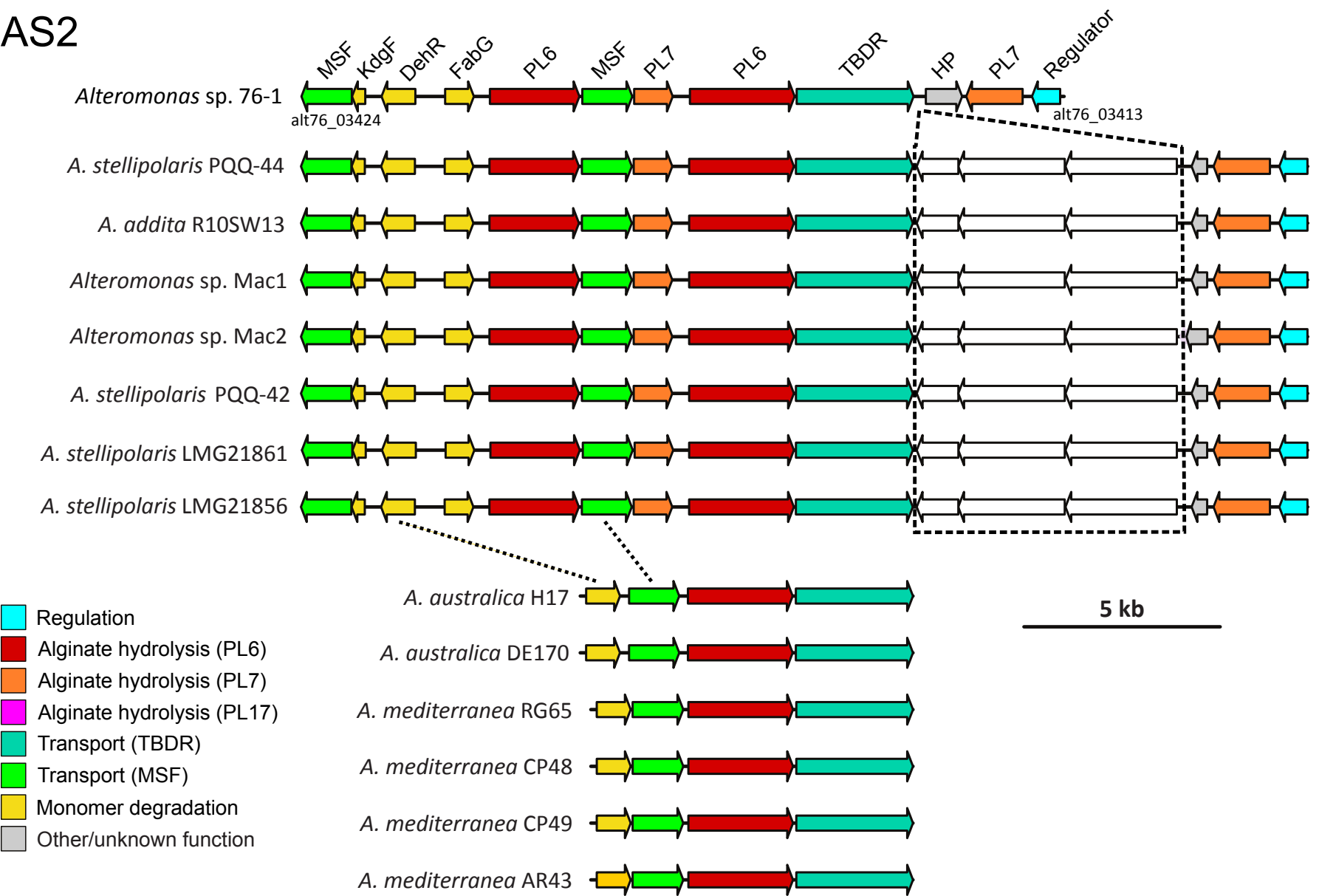

Supplement: FIGURE S3 — Detailed overview of AS1 and AS2 in Alteromonas sp. 76-1 and related strains. PL, polysaccharide lyase; TBDR, TonB-dependent receptor; MSF, major facilitator superfamily transporter; KdgF, protein for uronate linearization; DehR, 4-deoxy-L-erythro-5-hexoseulose uronate (DEH) reductase; FabG, oxidoreductase with similarity to DEH reductase; Nramp, metal-ion transporter; HP, protein of unknown function. Numbers below clusters designate IMG locus tags. [file Image_3.pdf]
